# Far-UV circular dichroism signatures indicate fluorophore labeling induced conformational changes of penetratin

**DOI:** 10.1007/s00726-022-03149-1

**Published:** 2022-03-18

**Authors:** Ferenc Zsila

**Affiliations:** Institute of Materials and Environmental Chemistry, Research Centre for Natural Sciences, P.O. Box 286, 1519 Budapest, Hungary

**Keywords:** Circular dichroism, Exciton coupling, Fluorophore labeling, Penetratin, Peptide conjugates, Rhodamine B

## Abstract

Fluorescent labeling is a broadly utilized approach to assess in vitro and in vivo behavior of biologically active, especially cell-penetrating and antimicrobial peptides. In this communication, far-UV circular dichroism (CD) spectra of penetratin (PEN) fluorophore conjugates reported previously have been re-evaluated. Compared to the intrinsically disordered native peptide, rhodamine B and carboxyfluorescein derivatives of free and membrane-bound PEN exhibit extrinsic CD features. Potential sources of these signals displayed above 220 nm are discussed suggesting the contributions of both intra- and intermolecular chiral exciton coupling mechanisms. Careful evaluation of the CD spectra of fluorophore-labeled peptides is a valuable tool for early detection of labeling-provoked structural alterations which in turn may modify the membrane binding and cellular uptake compared to the unconjugated form.

Circular dichroism (CD) spectroscopy is a special form of the commonly employed conventional UV/Vis absorption spectroscopy measuring the difference in the absorption between left- and right-handed circularly polarized light. This method is well suited to study the secondary structure of proteins and peptides since their typical structural components give rise to discernible spectral patterns in the far-UV region (170–250 nm) which are associated with the optically active n–π* and π–π* transitions of the amide chromophores (Toniolo et al. 2012). The most intense bands belong to the α-helical conformation and are displayed as two distinct negative maxima near 222 and 208 nm and a strong positive peak around 190 nm. The 222 nm band is of *n*–π* origin, whereas the shorter wavelength peaks are assigned to the exciton split components of the π – π* transitions (Woody 2005). The characteristic features of the β-sheet CD spectrum consist of a negative *n*– π* band near 217 nm and a positive–negative CD couplet centered at ~ 195 and ~ 175 nm, respectively. It is to be noted that the classical concept of the amide–amide exciton coupling model used for prediction the secondary structure of polypeptides has recently been challenged. Time-dependent density functional calculations complemented with CD and UV spectroscopic evaluation of cationic tripeptides suggested the contribution of multiple electronic transitions which involve not only molecular orbitals of the amide bonds but of the side chains and hydration water shell as well (Kumar et al. 2019, 2020).

The random coil (disordered) state of polypeptides produces a main negative CD band around 197–200 nm (Woody 2010; Toniolo et al. 2012). Disordered sequences are especially abundant among amphiphilic peptides showing pronounced affinity to lipid membranes (Latendorf et al. 2019; Yacoub et al. 2017). Antimicrobial (AMP) and cell-penetrating peptides (CPPs) are the most prominent representatives of such natural substances (Avci et al. 2018; Di Somma et al. 2020). They are relatively short (5–40 amino acids), predominantly cationic peptides. In many instances, they are unstructured in aqueous solution but readily adopt α-helical or β-sheet conformation upon interaction with lipid bilayers (Aisenbrey et al. 2019).

In order to assess membrane binding, cellular uptake and intracellular distribution of AMPs and CPPs, the peptide chain is often conjugated with an organic fluorophore to ensure sensitive fluorescent detection. However, labeling with bulky, aromatic dyes like 5(6)-carboxyfluorescein (CF), rhodamine B (RhB) and related compounds may alter physico-chemical properties of the peptides and also their biomolecular interactions compared to the native, unconjugated form (Cavaco et al. 2020; Seisel et al. 2019; Birch et al. 2017). Employing complementary biophysical methods, Hedegaard et al. evaluated how does fluorophore conjugation affect the interaction of penetratin with model membranes (Hedegaard et al. 2018). Penetratin (PEN) is a 16 residues long, widely utilized CPP derived from the homeodomain of *Drosophila antennapedia* (RQIKIWFQNRRMKWKK) (Dupont et al. 2015). Its N terminus was conjugated to six different fluorophores and the secondary structure of native PEN and its conjugates was compared by CD spectroscopy in the absence and presence of POPC/POPG (80:20) liposomes. The results, however, have been discussed superficially and no attention has been paid to some prominent spectral modifications. In line with previously reported data, the far-UV CD profile of native PEN measured in Tris–HCl buffer shows a lone trough at ~ 199 nm which is typical of a highly disordered state (Woody 2010). For the RhB conjugate, the λ_min_ was shifted above 200 nm and a zero cross-over point appeared around 193 nm. These alterations are diagnostic to a shift in the dynamic conformational equilibrium towards the helically folded species (Zsila et al. 2019). However, RhB-PEN exhibits an additional, clearly discernible negative Cotton effect (CE) with a maximum at 233 nm that is completely lacks from the CD spectrum of the unconjugated peptide (Fig. 1A).

**Fig. 1:**
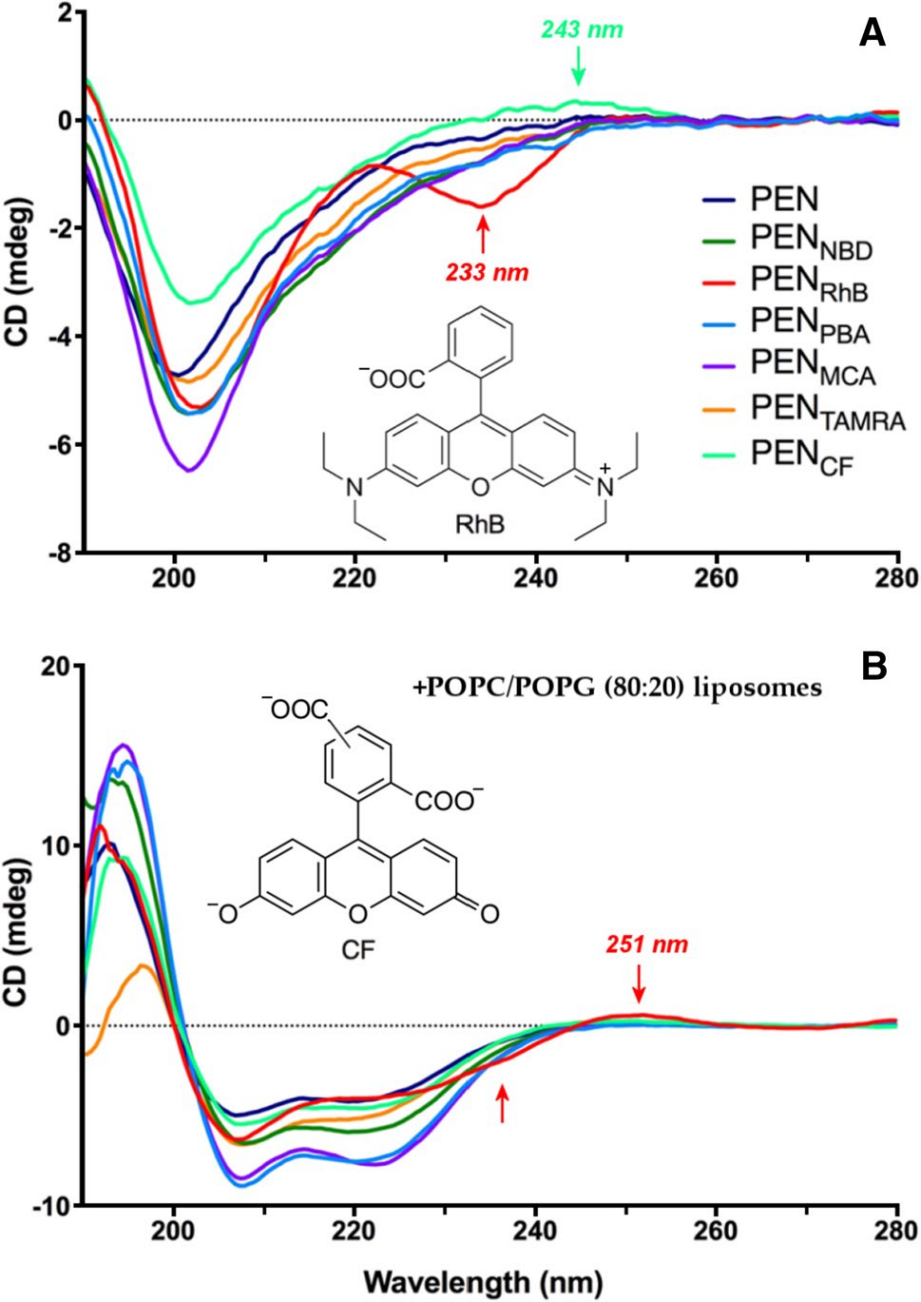
Far-UV CD spectra of 20 µM penetratin (PEN) and its six fluorophore conjugates as measured in Tris–HCl buffer (10 mM, pH 7.4) in the absence (**A**) and presence (**B**) of POPC/POPG (80:20%) liposomes with a total lipid concentration of 2 mM. Arrows denote induced CD bands of rhodamine B (RhB) and 5(6)-carboxyfluorescein (CF) conjugates of PEN. TAMRA: 5(6)-carboxytetramethylrhodamine, NBD: N-(7-nitro-2,1,3-benzoxadiazol-4-yl)glycine, MCA: (7-methoxycoumarin-4-yl)acetic and PBA: 1-pyrenebutyric acid. Adapted from (Hedegaard et al. 2018)

Besides similar spectral indicators of growing helical contribution, CF-labeled PEN derivative also displayed a novel CD feature as a broad, positive ellipticity band above 230 nm (Fig. 1A). For the other conjugates, however, no any spectral traces of new CD bands could be noted. The UV spectrum of free RhB shows several absorption bands in the far-UV window relevant to secondary structure determination of peptides and proteins (Fig. 2A).

**Fig. 2:**
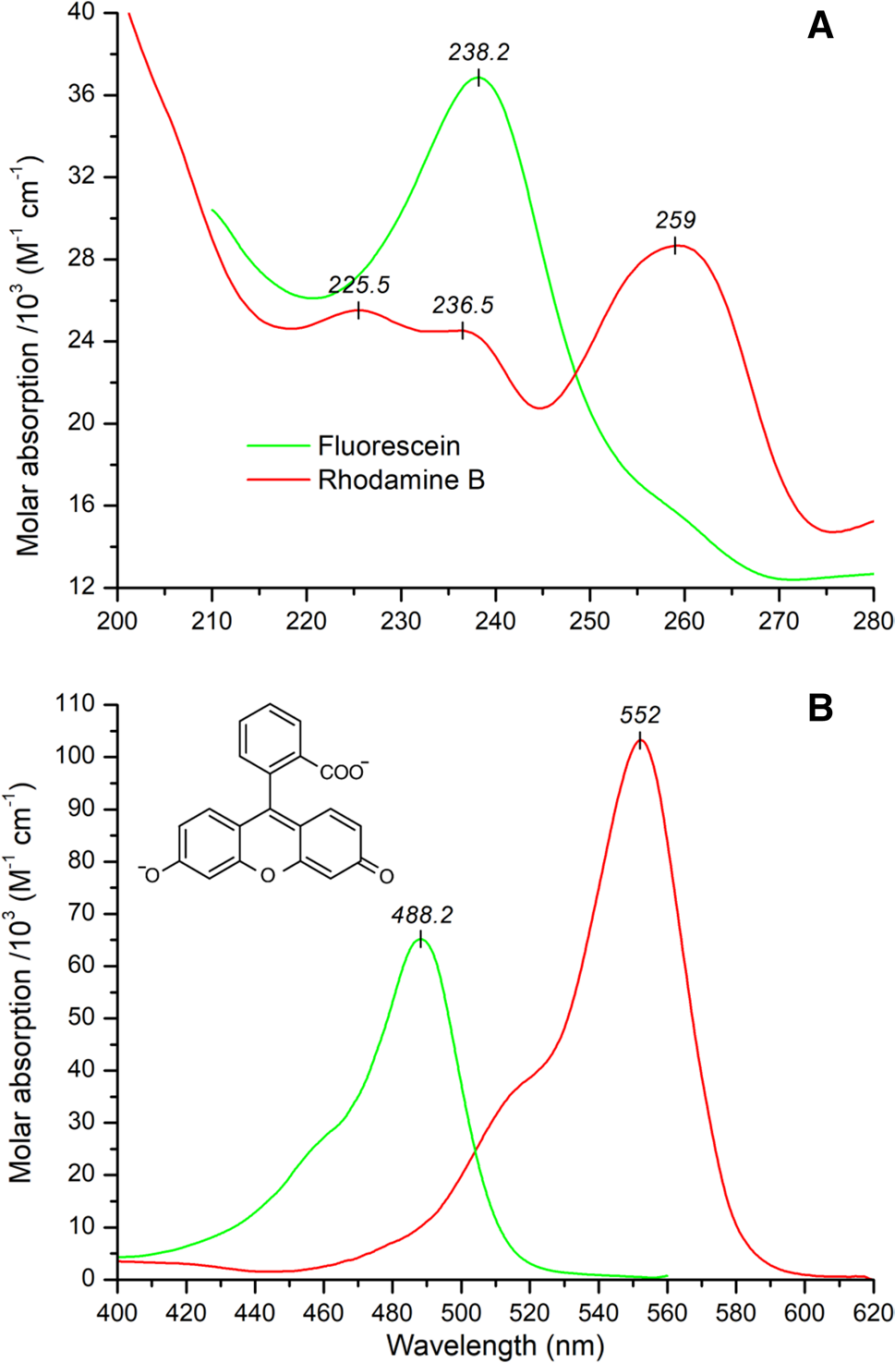
Far-UV (**A**) and visible (**B**) absorption spectra of rhodamine B and fluorescein disodium salt measured in 10 mM phosphate buffer at pH 7.0 and 7.4, respectively. Adapted from (Zsila and Iwao 2007; Zsila 2011). The chemical structure of the dianionic form of fluorescein is shown

Fluorescein also has an intense UV peak at 238 nm and a shoulder around 260 nm. Therefore, spectral positions of the extra CD signatures (Fig. 1A) suggest their association with the π– π* transitions of the achiral fluorescent dyes which gained CD activity due to their covalent attachment to the chiral peptide chain. Most likely, the mechanism of CD induction involves intramolecular chiral exciton coupling between the respective electronic transition dipole moments of the fluorophore and proximal aromatic residues (Zsila 2011; Zsila and Iwao 2007; Tetin and Linthicum 1996). PEN contains two tryptophans at the sixth and the fourteenth position. The indole ring has an intense absorption band at ~ 219 nm (λ_max_ ≈ 35 000 M^−1^ cm^−1^ (Nishino et al. 2002) that favors non-degenerate coupled oscillator interaction with energetically close-lying far-UV π–π* transitions of CF and RhB (Fig. 2A) (Grishina and Woody 1994). For an optically active dye-Trp exciton coupling, a chiral spatial orientation of the planar xanthene moiety relative to the indole ring is required. In other words, the label in CF-PEN and RhB-PEN conjugates adopts a preferred steric orientation related to a Trp residue stabilized by non-covalent intrapeptide interactions. It is worth to mention, that differences in internalization levels reported recently between tryptophan-rich TAMRA-CPP conjugates have in part been attributed to intramolecular dye-Trp π -stacking interactions (Seisel et al. 2019). Such kind of structural modification also affects the folding as well as the membrane binding properties of the conjugate. In concordance with this, particularly high membrane disturbance was observed for RhB-PEN compared to the native peptide (Hedegaard et al. 2018). It is worth to mention that several proteins and peptides have anomalous far-UV CD spectrum exhibiting a positive or a negative CD band in the 225–235 nm region (Woody 1978; Vuilleumier et al. 1993; Clark et al. 1996). Apart from the presence of disulfide bonds, these signals stem from intramolecular aromatic–aromatic side chain or aromatic side chain–amide bond exciton interactions. Accordingly, in RhB-PEN the Trp residues alone may be responsible for the negative CE at 233 nm but in that case this extra signal should be observed for the unconjugated peptide as well. The native PEN, however, does not show such a CD peak (Fig. 1A) suggesting the decisive role of fluorophore-Trp exciton coupling in RhB-PEN and CF-PEN conjugates.

Noticeably, the induced CE of RhB-PEN prevails even in its membrane-bound, helical form (Fig. 1B). Due to the spectral overlap with the *n*-π* band of the folded peptide chain, the 233 nm negative peak can be observed as a shoulder only. What is more, an additional positive extrinsic CE can be recognized between 245 and 260 nm (Fig. 1B). This may reflect some sterical re-adjustment of the RhB unit prompted by the membrane binding caused helical conversion of the peptide backbone that allows chiral perturbation of an additional π–π* transition of the dye. Alternatively, this new CD band may come from the self-association of RhB-PEN in the lipid bilayer which is crucial for the capability of AMPs and CPPs to affect microbial membranes (Pirtskhalava et al. 2021). Due to the tight packing of the peptide chains, intermolecular exciton coupling may also occur between RhB labels positioned close to each other inside the self-assembly. This type of exciton interaction is characterized by two CEs with opposite signs, which reflects the intermolecular steric disposition between the two chromophores (Boiadjiev and Lightner 2005).

Unfortunately, Hedegaard et al. did not extend their CD scans to the longer wavelength range of the spectrum where xanthene dyes exhibit much stronger light absorption. Besides some lower intensity peaks between 280 and 400 nm, CF and RhB display a highly intense (ε ≈ 10^5^) band centered around 490 and 553 nm, respectively (Fig. 2B). CD spectroscopic studies of antibody (Tetin and Hazlett 2000; Athey and Cathou 1977), avidin (Zsila 2011) and serum protein (Zsila and Iwao 2007) binding of fluorescein and RhB indicated that dye-aromatic residue exciton coupling is the major source of multiple, extrinsic CEs observed in both the UV and visible spectra of these compounds. Therefore, it is reasonable to assume that xanthene fluorophore conjugates of PEN might give rise to induced CD signals not only in the far-UV but the near-UV and visible absorption spectrum as well.

In summary, these observations emphasize the need for careful examination of CD curves of fluorophore–peptide conjugates either in free and membrane-bound state. It may allow the early recognition of label-aromatic side chain intramolecular and label-label intermolecular interactions which can affect the conformational as well as membrane insertion properties of conjugates compared to the native peptide.

## Abbreviations

AMP: Antimicrobial peptide
CD: Circular dichroism
CE: Cotton effect
CF: 5(6)-Carboxyfluorescein
CPP: Cell-penetrating peptide
PEN: Penetratin
RhB: Rhodamine B
